# Nasopharyngeal B-cell lymphoma with pan-hypopituitarism and oculomotor nerve palsy: a case report and review of the literature

**DOI:** 10.1186/s12902-020-00644-y

**Published:** 2020-11-03

**Authors:** Maryam Zahedi, Reyhane Hizomi Arani, Maryam Tohidi, Shirin Haghighi, Masoud Mehrpour, Farzad Hadaegh

**Affiliations:** 1 Prevention of Metabolic Disorders Research Center, Research Institute for Endocrine Sciences, Shahid Beheshti University of Medical Sciences, No. 24, Parvaneh Street, P.O. Box: 19395-4763, Tehran, Velenjak Iran; 2 Taleghani Hospital, Shahid Beheshti University of Medical Sciences, Tehran, Iran; 3 Stroke center, Firoozgar General Hospital, Tehran University of Medical Sciences, Tehran, Iran

**Keywords:** Nasopharyngeal lymphoma, Hypopituitarism, Central diabetes Incipidious (CDI), Oculomotor nerve palsy, Case report

## Abstract

**Background:**

Primary nasopharyngeal lymphoma (NPL) is a very rare tumor of Waldeyer ring (WR) lymphoid tissue. It is challenging to differentiate lymphoma infiltration of pituitary from a pituitary adenoma, meningioma infiltration, and other sellar lesions to plan a suitable treatment strategy. We presented for the first time a unique case of NPL with an unusual presentation of oculomotor nerve palsy associated with pan-pituitary involvement in a diabetic patient.

**Case presentation:**

A 64-year old diabetic woman with no previous history of malignancy presented with intermittent diplopia for about the last nine months. Severe headache, left eye ptosis and hypoglycemic episodes were added to her symptoms after a while. Further complaints include generalized weakness, loss of appetite, generalized musculoskeletal pain, and 6–7 kg weight loss within six months. Her family history was unremarkable. Physical examinations of eyes indicated left eye 3rd, 4th, and 6th nerve palsy. But, she was not anisocoric, and the pupillary reflexes were normal on both eyes. No lymphadenopathy, organomegaly and other abnormalities were found. Magnetic resonance imaging (MRI) showed a heterogeneous enhancement in the seller and suprasellar regions, enlargement of the stalk, parasellar dural enhancement and thickening of the sphenoid sinus without bone erosion. Also, both cavernous sinuses were infiltrated and both internal carotid arteries were encased by the neoplastic lesion. It suggested an infiltrative neoplastic lesion which compressed the cranial nerves. Pituitary hormone levels assessment indicated a pan-hypopituitarism. Following nasopharyngeal mucosal biopsy, the immunohistochemistry (IHC) findings revealed a low-grade non-Hodgkin’s B-cell lymphoma. Systemic workup, including cerebrospinal fluid (CSF) studies, bone marrow aspiration, chest and abdominopelvic high-resolution computed tomography (HRCT) indicated no other involvement by the lymphoma. After chemotherapy courses, central adrenal insufficiency, partial central diabetes incipidious (CDI) and central hypothyroidism have been resolved. To our best knowledge, we found 17 cases of NPL with cranial nerve palsy, 1 case of NPL with pan-hypopituitarism and no NPL case with both cranial nerve palsy and pituitary dysfunction.

**Conclusions:**

The incidence of cranial neuropathy in patients with diabetes should not merely be attributed to diabetic neuropathy without further evaluation.

## Background

Nasopharyngeal lymphoma (NPL) is a rare malignancy with extranodal lymphoid proliferation [1]. NPL is classified into Hodgkin lymphoma and non-Hodgkin lymphoma (NHL). NHL lymphoma accounts for 86 to 90% of all lymphoma cases [2, 3]. Lymphoid tissues of the palatine tonsils, soft palate, nasopharynx, oropharyngeal wall, and base of the tongue is known as the Waldeyer’s ring [1]. Previous studies have indicated that less than 10–18% of NHL cases involve the Waldeyer’s ring [1, 4], which about 35–37% of them were at the nasopharyngeal site [1]. In most cases, NPL appears with nasal manifestations, including epistaxis, nasal obstruction, and purulent rhinorrhea. However, it can present with a neck mass, headache, and B symptoms (i.e., weight loss, night sweats, and fever), less commonly [5].

On the other hand, 10–15% of intracranial neoplasms were attributed to pituitary tumors [6]. It has been reported that pituitary adenoma and meningioma are the most common tumors which can involve the pituitary gland. However, it is a rare site for diffuse malignant disease and metastasis [7]. The pituitary gland can be involved by metastatic lesions via the skull base, hematogenous, or meningeal spread [8]. According to a recent systematic review, the pituitary metastases (PM) are uncommon, accounting for 0.4% of intracranial metastases [9]. Almost every cancer is reported having a potential source for sellar metastasis. Lung and breast neoplasms are responsible for two-thirds of PM. The frequency of NHL involving the hypothalamus-pituitary axis is < 0.5% among PM [10]. A systematic review in 2015 stated that the most common symptom among all reported PM cases is diabetes insipidus. Anterior hypopituitarism (39.66%), visual deterioration (41.38%), cranial nerve palsies (41.38%) and headaches (32.76%) were the other symptoms which were reported. As symptomatic PM can be closely mimic a pituitary adenoma, the presentations of diabetes insipidus and/or cranial neuropathies could suggest PM rather than pituitary adenoma, especially in a rapidly developed courses, and in patients over 50 years old. Moreover, some studies have suggested that the presence of bony erosion without sellar enlargement indicates PM more than a pituitary adenoma [11]. PM has a poor prognosis and the management of patients with PM is palliative. The diagnosis of such malignancies is challenging because patients mostly presented nonspecific signs that could overshadow symptoms of hypopituitarism or diabetes insipidus, so the diagnosis is ultimately confirmed by histopathology [9, 12]. Sellar masses are rarely constituted by infiltrative neoplasm (such as lymphoma), inflammatory and granulomatous diseases of the pituitary [13]. Therefore, in a patient with lymphoma, it is essential to differentiate lymphoma infiltration of the pituitary from benign lesions to plan an appropriate treatment strategy.

## Case presentation

A 64-year old woman with a history of type 2 diabetes mellitus for more than thirty years and no previous history of malignancy presented with intermittent diplopia for about the last nine months, especially while going down the stairs. Diplopia was gradually increasing frequency and intensity in the previous few months. During the previous two months, the patient developed a severe headache, left eye ptosis, and hypoglycemic episodes. Further complaints include generalized weakness, loss of appetite, generalized musculoskeletal pain, and 6–7 kg weight loss within six months. Her hemoglobin A1C levels were around 7% in prior visits. Her family history was unremarkable.

**Fig. 1:**
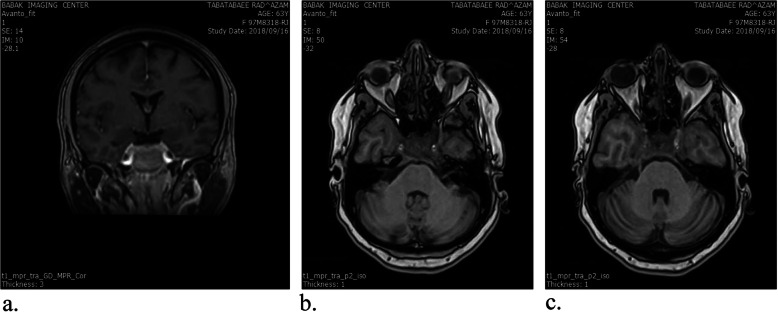
Coronal and sagittal planes of Pituitary and hypothalamus magnetic resonance imaging ± Gadolinium at baseline

In our initial physical examinations, she was obese (body mass index =34 kg/m^2^), and her blood pressure was 120/80 mmHg. No lymphadenopathy and organomegaly were found. No other abnormalities were noted on physical examinations except for left eye ptosis (3rd, 4th, and 6th nerve palsy). But, she was not anisocoric, and the pupillary reflexes were normal on both eyes. The pupils were round and equal and reacted to light consensually and directly. Visual acuity was normal in both eyes. The other examinations of cranial nerves were normal. Magnetic resonance imaging (MRI) of the hypothalamus and pituitary indicated a heterogeneous enhancement of the sellar and suprasellar regions, enlargement of the stalk, parasellar dural enhancement with the involvement of both cavernous sinuses and internal carotid arteries, and thickening of the sphenoid sinus without any bone erosion. It was not possible to differentiate between pituitary tumor and infiltrated nasopharyngeal region. It suggested an infiltrative neoplastic lesion that compressed left III, IV and VI cranial nerves. Also, the thickening of the sphenoid sinuses and nasopharyngeal regions was seen (Fig. 1).

Complete blood cell testing showed leukopenia and thrombocytopenia. As per whole blood count, red blood cell (RBC) and platelet counts were 4.69 Mil/ mm^3^ and 88,000/mm^3^, respectively. Also, white blood cell count (WBC) was 3100/mm^3^ (consist of 44% lymphocytes, 45% neutrophils, 8.7% monocyte, 1.6% eosinophil and 0.7% basophil). Hemoglobin and hematocrit were 12.9 g/dl and 37.6%, respectively.

To evaluate the patient for infiltrative disease, i.e., lymphoma, sarcoidosis, and tuberculosis, we tested serum lactate dehydrogenase (LDH) and angiotensin-converting enzyme (ACE) levels, which were reported in the normal range (LDH: 353 (230–460 U/L) and ACE: 51.1(8–52 U/L)). Also, the purified protein derivative (PPD/tuberculin) test was negative.

To rule out immunological diseases that could infiltrate cavernous sinuses and pituitary gland area (e.g., Wegener’s granulomatosis, Ig G4 related disease), immunologic assays were done. All serum immunology assays except perinuclear anti-neutrophil antibodies (P-ANCA or anti-MPO) were within their reference values i.e. immunoglobulin G (IgG): 1055 (700–1600 mg/dL), Ig A: 208 (70-400 mg/dL), Ig M: 45 (40–230 mg/dL), Cytoplasmic anti-neutrophil antibodies (C-ANCA or Anti-PR3): 4.9 (Negative: < 10 U/mL), and IgG4: 550.3 (39–864 mg/L). The result of P-ANCA was 23.5 U/mL (Negative: < 10). The bone marrow aspiration and biopsy were performed according to bi-cytopenia that showed cellular marrow without atypia.

Laboratory evaluation of hypothalamic and pituitary axis revealed a pan-hypopituitarism i.e. free T4: 0.6 (0.7–2.5 ng/dL), free T3: 0.18 (0.2–0.5 ng/dL), thyroid-stimulating hormone (TSH): 0.47 (0.3–4.2 μIU/mL), cortisol at 8 am: 3 (5–23 μg/dL), adrenocorticotrophic hormone (ACTH): 24.78 (7.2–64 pg/mL), luteinizing hormone (LH): 1.2 (8.2–40.8 IU/L), follicle-stimulating hormone (FSH): 5.3 (35–153 IU/L), prolactin: 9.7 (2.1–17.7 ng/mL), and insulin like growth factor-1 (IGF-1): 24 (33–220 ng/mL).

In cerebrospinal fluid (CSF) analyses, protein and LDH were elevated (Table 1). CSF cytology examination showed a few small lymphoid cells with irregular nuclei.

**Table 1:** Cerebrospinal fluid (CSF) analyses

CSF components	Results	Normal value
**Sugar (mg/dL)**	113	**40–70**
**Protein (mg/dL)**	183.8	**15–45**
**LDH (U/L)**	71	**10% of the serum value (Serum level of LDH: 353 U/L)**
**WBC (/Cumm)**	80	**–**
**PMN (number)**	5	**–**
**MN (number)**	80	**–**
**RBC (/cumm)**	60	**–**
**CSF culture**	No growth	**–**
**VDRL**	No reactive	**–**
**MTB/NTM DNA PCR**	Undetectable	**–**

*LDH* Lactate dehydrogenase, *WBC* White blood cell, *PMN* Polymorphonuclear leukocyte, *MN* mononuclear leukocyte, *RBC* Red blood cell, *VDRL* Venereal disease research laboratory, *MTB/NTM DNA PCR Mycobacterium tuberculosis* / nontuberculous mycobacteria DNA polymerase chain reaction

**Fig. 2:**
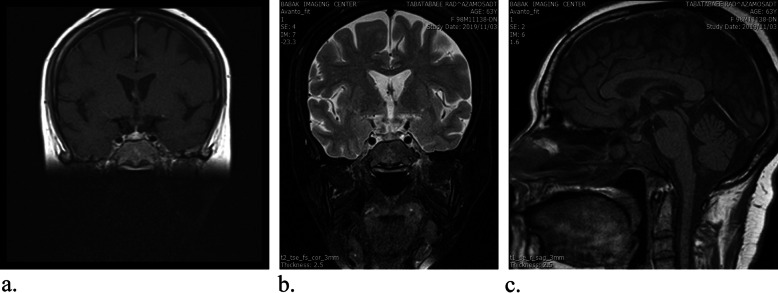
Histopathologic features of nasopharyngeal tissue biopsies: **a** and **b** H&E staining of nasopharyngeal mucosal tissue infiltrated by atypical lymphocytes; **c** Positive IHC for CD20; **d** IHC for ki67. H&E, Hematoxylin-Eosin; IHC, Immunohistochemistry

Pathologic findings of tissue biopsy of thickened nasopharyngeal mucosa reported a low-grade lymphoma. Cell immunohistochemistry (IHC) were positive for CD20 and Bcl-2 in most lymphoid cells (B-cells). Moreover, CD3 and CD5 were positive, and CD10 was negative in some lymphoid cells (T-cells). Also, CD-23 and cyclin D1 were negative, and cell proliferation index Ki-67 was about 10%. These findings revealed a low-grade B-cell lymphoma (Fig. 2).

Chest and abdominopelvic high-resolution computed tomography (HRCT) indicated no abnormalities and lymphadenopathy. So we concluded that the final diagnosis for the current patient was a primary lymphoma originated from the nasopharyngeal mucosa by spreading the upwards areas, including both cavernous sinuses, sellar, and suprasellar regions.

**Fig. 3:**
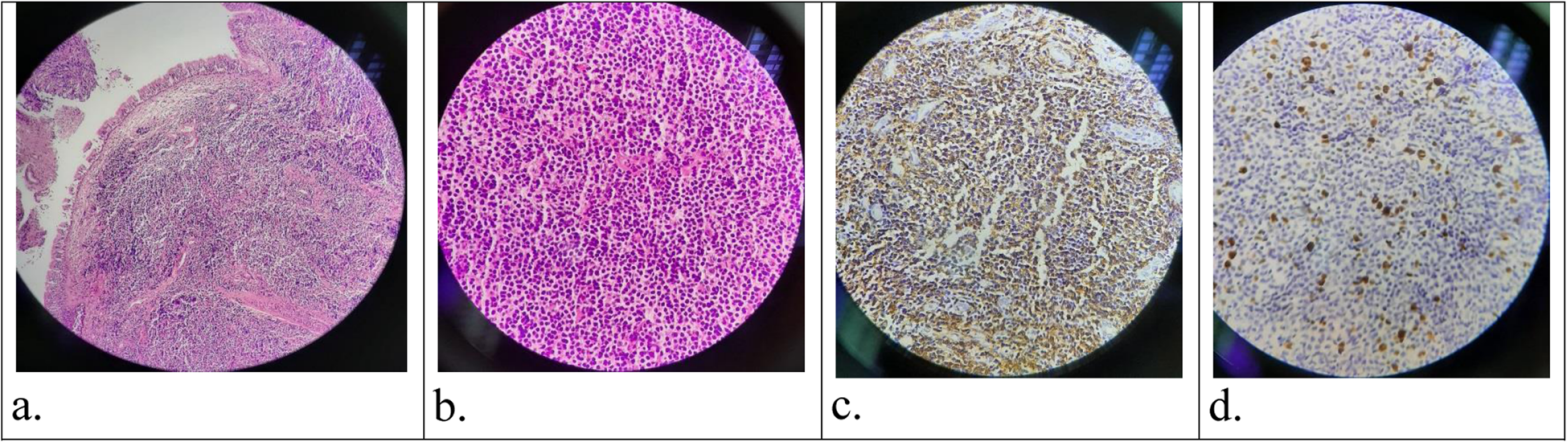
Coronal and sagittal planes of Pituitary and hypothalamus magnetic resonance imaging ± Gadolinium after completing chemotherapy

She received six courses of chemotherapy with CHOP: cyclophosphamide, doxorubicin hydrochloride, vincristine sulfate, and dexamethasone plus Rituximab. Oral prednisolone (7.5 mg daily) and levothyroxine (50 μg daily) were prescribed simultaneously with chemotherapy, due to her pan-hypopituitarism. A few days after prednisolone usage, ptosis dramatically improved. MRI enhancement in the sellar and suprasellar regions and both cavernous sinuses were largely eliminated after the last chemotherapy course (Fig. 3). Besides, hematological defects were improved significantly.

One month after treatment with prednisolone, the patient complained about polyuria and nocturia. Partial central diabetes insidious (CDI) was diagnosed based on more than 3 l 24-h urine volume, mild hypernatremia (Na: 147 meq/L) and low urine osmolality (urine specific gravity was 1.005 whereas the urine specific gravity in the first evaluation was 1.010). After chemotherapy courses central adrenal insufficiency, partial CDI and central hypothyroidism have been resolved.

### Search strategy for literature review

We searched PubMed for articles published from Jan 1, 1990, to Aug 1, 2020, using the search terms “nasopharyngeal lymphoma”, “non-Hodgkin’s lymphoma of the nasopharynx”, “nasopharyngeal B-cell non-Hodgkin’s lymphoma”, “nasopharyngeal Hodgkin’s disease” in combination with the terms “pan-hypopituitarism”, “pituitary dysfunction”, “cranial nerves palsy”, “multiple cranial nerve palsy”, “oculomotor nerve palsy”, “isolated oculomotor nerve palsy”, “multiple cranial nerve dysfunction”,” III cranial nerve palsy”, “IV cranial nerve palsy”, “VI cranial nerve palsy”, “third cranial nerve palsy”,“4th cranial nerve palsy”,“6th cranial nerve palsy”. Articles published in English were included. We focused mostly on articles from case reports or case series.

## Discussion and Conclusions

We have described a woman with type 2 diabetes mellitus and nasopharyngeal B-cell lymphoma infiltration of both cavernous sinuses and pituitary gland, who presented with the left eye ptosis (3rd, 4th, and 6th nerve palsy), severe headache, and pan-hypopituitarism.

Clinical presentations in our patient (i.e., hypopituitarism, headaches, and visual disturbances) could suggest the infiltration of the pituitary gland by lymphoma, leukemia, and metastasis to the pituitary. In our patient, the left eye oculomotor nerve palsy suggested two main differential diagnoses as diabetic cranial neuropathy or cavernous sinuses involvement.

A comprehensive review on the management of III nerve palsy suggested that when a patient presents with an acute onset of unilateral limitation of an eye, the defect should be categorized to “complete or partial” and “with or without the involvement of the pupil” to come to a diagnosis. Pupil-sparing in old patients with known systemic vascular disease can suggest ischemic mononeuropathy as a common cause [14]

The adjacent cavernous sinus infiltration involving nerve III, IV, and VI, usually induces cranial nerve palsy in decreasing order of frequency. 6th nerve palsy is relatively uncommon because it is well sheltered in the cavernous sinus [7]. In diabetic neuropathy, multiple cranial nerve palsies are extremely rare, and pupillary reflex usually spared because the ischemic lesion is confined to the core of the nerve and does not affect peripherally situated pupillomotor fibers [15]. Although diabetic neuropathy is the most common cause of third nerve palsy, it is advisable to perform a brain MRI to exclude other causes of oculomotor nerve palsy [16].

On the other hand, poor glycemic control or rapid treatment of hyperglycemia could increase the risk of diabetic neuropathy [17]. It may have an acute onset resulting from ischemic infarction of the vasa nervorum [18]. Diabetic neuropathy was less suggested in our patient due to well-controlled diabetes, the chronic and insidious presentation of the symptoms, and multi-neuropathy involvement.

Sato et al. stated that isolated oculomotor nerve palsy was most frequently associated with the large B-cell lymphoma cell type. Pupil sparing oculomotor nerve palsy suggests infarction of the oculomotor nerve, as is commonly observed in patients with diabetes mellitus; despite this, there was no infarction of the oculomotor nerve on histological examination in the reported cases with lymphoma and isolated oculomotor palsy [19] These findings suggest that whether the pupil is involved or spared may depend on damage to the pupilomotor fibers in the oculomotor nerve by infiltration or compression by lymphoma. Moreover, as acknowledged by Brazis, compressive cavernous sinus lesions might spare the pupil “because they often involve only the superior division of the oculomotor nerve that carries no pupillomotor fibers, or the superior aspect of the nerve anterior to the point where the pupillomotor fibers descend in their course near the inferior oblique muscle” [15]

Excluding neoplastic disorders, other etiologies of multiple cranial nerve palsy include infections (e.g., *Mycobacterium tuberculosis*), inflammatory diseases (e.g., sarcoidosis, vasculitis, Wegener’s granulomatosis, amyloidosis, connective tissue disease, rheumatoid arthritis), vascular disease (e.g., diabetes, aneurysm, carotid artery dissection, sickle-cell disease), bone disease (e.g., Paget’s disease) and trauma (e.g., closed head injury) [20].

Regarding the source of the lymphoma, pituitary lymphoma is very rare, and there is no report for extra-sellar spreading in literature till now [21]. On the other hand, malignancy of WR lymphoid tissue and primary involvement of nasopharyngeal is an uncommon tumor that includes a small part of NHL and has the potential to infiltrate the adjacent tissues [1]. So, in this case nasopharyngeal lymphoma was more probable than primary CNS lymphoma.

A few studies are reporting the clinical characteristics of NH lymphomas [22, 23]. Hsueh et al. reported that in 35 cases of NPL during 22 years’ follow-up with the average age of 59.6 years, WBC of 12,992/mm^3^, LDH of 337.7 U/L, and the meantime from initial symptoms to diagnosis of 2.6 months, neck lymph nodes involvement or other distant involvements were detected in less than a third of patients at the time of diagnosis. Also, 14.3% of the patients were presented with B symptoms. Diffuse large B cell lymphoma was the most common pathological diagnosis of nasopharyngeal (*n* = 17), followed by NK/T cell lymphoma (*n* = 9). Extranodal marginal zone lymphoma of mucosa-associated lymphoid tissue, mantle cell lymphoma, and small lymphocytic lymphoma was the other pathologic diagnoses [5]. To compare our patient with Hsueh et al. study, our patient had leukopenia and a longer duration from initial symptoms (i.e., diplopia) to diagnosis (about 9 months) and similar age and LDH levels. Pathological findings in our patient were compatible with mantle zone lymphoma, which was one of the lesser-known cases in NH lymphomas. Our patient’s presentations were noteworthy due to her pituitary and cavernous sinus involvement, while she had no remarkable B-symptoms and nasal involvement. Unusual manifestations of a rare disease led to a prolonged diagnosis.

NHL of the nasopharyngeal region is usually invasive and has a strikingly poor prognosis than other extranodal lymphomas [24]. Also, localized disease and low-grade NPL are associated with a better prognosis [22]. Moreover, B symptoms have been reported to be associated with poor prognosis [25]. Our patient suggested having a relatively good prognosis due to her localized and low-grade disease.

There are limited data regarding epidemiologic and treatment outcomes of NH lymphoma [5]. The treatment of localized disease (stage I, II and non-bulky disease) with activity index less than 2 and normal LDH level is based on the CHOP regimen (3 to 4 cycles) plus Rituximab (if CD20 positive in immunochemistry). Based on Allam et al. study, More than 80% of patients may be successfully treated by this regimen [22]. Our patient responded to chemotherapy and resolved her hematological defects.

Glucocorticoids act as a down-regulatory signal to suppress arginine vasopressin (AVP (and corticotropin-releasing hormone (CRH (secretion via negative feedback loops, respectively. In patients with hypocortisolism, glucocorticoid deficiency stimulates CRH, and therefore AVP release. So glucocorticoid replacement could increase free water excretion and unmask the concomitant CDI [26] as showen in our patient. Although pituitary dysfunction was improved in most cases, complete recovery occurred less frequently [27]. Adrenal insufficiency, central hypothyroidism and CDI have been resolved in our patient.

To our best knowledge, we found 17 cases of NPL with cranial nerve palsy, 1 case of NPL with pan-hypopituitarism and no NPL case with both cranial nerve palsy and pituitary dysfunction as showed in Table 2 [28–35].

**Table 2:** Demographics, clinical features, treatment and outcomes of nasopharyngeal lymphoma patients with presentation of cranial nerve palsy OR pituitary dysfunction

Author,[Reference]	Age (years),gender	Clinical presentation	Other clinical features	Neurological deficits	Radiological findings	Histological diagnosis	Treatment and outcome
Mohammadianpanah, [28]	1 patient (Not described)	A bulky primary tumor and regional cervical lymphadenopathy was defined as a size ≥5 cm in its maximal diameter	Nasal obstruction and dysphagia	3th and 6th cranial nerve palsy	Extension to the adjacent structures; most commonly into the nasal cavity	Non-Hodgkin*’*s lymphoma of the nasopharynx	CHOP regimen and radiation therapy
Lopes da Silva, [29]	28,Male	Diplopia	Proptosis of the eye	Involvement of the trigeminus nerve	Infiltration of the posterior-inferior side of the right orbit	B lymphoblastic lymphoma of the nasopharynx	Complete remission of the disease after 2 years.
KAY, [30]	19, Male	Ulceration of the soft palate and uvula	Blepharoptosis of the left eye, weakness of the right side of face, diplopia, and a funny taste.	6th nerve paralysis. A right peripheral 7th nerve palsy. Motor pupil defect on the right. 4th nerve palsy, partial 3th nerve palsy on the right	Chest x-ray shows hilar enlargement, nodular densities and cavitary lesions	Diffuse infiltrate of atypical lymphocyte in nasopharyngeal biopsy	Radiation therapy to the base of the brain. Dexamethasone and cyclophosphamide orally. Died shortly
Keane, [31]	3 patients (Not described)	Presence of severe weakness, atrophy or fasciculations, and deviation of the tongue on protrusion.		Twelfth-Nerve Palsy and 3th,5th, 6th10th, 11th may to impair	MR imaging evidence of a large nasopharyngeal mass spreads within the cavernous sinus and extend laterally into the neck	Nasopharyngeal lymphoma	Radiation
RIGGS, [32]	50, Male	Double vision, vertigo, and unsteady gait	External rectus muscle paralysis	Right 6th nerve palsy	Infiltration of the left cavernous sinus and dura over the Gasserian ganglion with malignant	Nasopharyngeal lymphoma	Died in 6 month
RIGGS, [32]	30, Male	Painless, rapidly growing mass on the left side of the neck, suddenly became blind in the left eye and developed	Ptosis on the left eye. Numbness of the left side of the face, dilated and fixed pupil on the left with limitation of all movement of the eyeball.	Left peripheral facial palsy with weakness of the right side of the face	Both cavernous sinuses and the left carotid artery was obliterated by the mass. Metastatic tumor was present in the lung and pancreas and cervical lymph nodes.	Nasopharyngeal lymphoma	X-ray therapy, died in 6 month
RIGGS, [32]	50, Male	Painful mass on the right side of the neck, pain and ptosis of the left lid		6th nerve palsy	The Gasserian ganglia were embedded in tumor, and neoplastic tissue obliterated the subdural space of both optics nerves.	Nasopharyngeal lymphoma	Died 31 month later
RIGGS, [32]	36, Male	Painless growth on the left side of the neck		Left peripheral facial palsy and 3,4,6,9,10 nerve palsy	A mass filled dorsum of the sella, petrous bone, and the adjacent sphenoid bone. Obliterated the cavernous sinus on this side and constricted the internal carotid	Nasopharyngeal lymphoma	2 years
RIGGS, [32]	34, Male	Pain and fullness in the throat for three months. Severe, constant pain in the right side of the face.	Weakness of the soft palate, and decreased hearing on the right. Weakness of the right sternocleidomastoid muscle.	Partial peripheral facial palsy	Destruction of the right middle fossa	Nasopharyngeal lymphoma	Died after 29 month
Van der Vliet [33]	47, Female	Pain in right middle ear and right-sided hearing loss and tinnitus. Loss of sensation of the right half of the tongue	The pupil of the left eye was larger than that of the right	Unilateral multiple cranial nerve dysfunction. (nerves V, VII, VIII, IX, X, and XII)	Effusion of the mastoid air cells and middle ear. Intracranial extension via the foramen ovale into Meckel’s cavity and in the hypoglossal canal	Nasopharyngeal B-cell non-Hodgkin’s lymphoma	CHOP chemotherapy, died early
Ingram [34]	5patients (male aged 4,10,4,15;female aged 9)	(Not described specifically)	(Not described specifically)	3th and 7th cranial nerve palsy	(Not described specifically)	Nasopharyngeal B-cell non-Hodgkin’s lymphoma	Radiation,3of them alive,2 of them died
Bunick [35]	47,Male	Diplopia, headache, Lethargy, hearing loss	Pan hypopituitarism, Low libido, coldness, loss of body hair	–	Skull X-ray -destruction of floor of sella	Nasopharyngeal Hodgkin’s lymphoma	MOPP Rdx, T4, T, GC as hormone treatment
Current case	64, Female	Intermittent diplopia. Severe headache, left eye ptosis, and hypoglycemic episodes	Pan hypopituitarism generalized weakness, generalized musculoskeletal pain, and 6–7 kg weight loss	Left eye 3rd, 4th, and 6th nerve palsy. But, she was not anisocoric and the pupillary reflexes were normal on both eyes	MRI showed a heterogeneous enhancement in the seller and suprasellar regions, enlargement of the stalk, parasellar dural enhancement and thickening of the sphenoid sinus without any bone erosion	low-grade non-Hodgkin’s B-cell lymphoma	CHOP chemotherapy. Oral prednisolone and levothyroxine. Central adrenal insufficiency,partial CDI and central hypothyroidism have been resolved.

*CHOP* Cyclophosphamide, doxorubicin hydrochloride, vincristine sulfate, and dexamethasone, *MRI* Magnetic resonance imaging, *CDI* Central diabetes insidious

In conclusion, we presented for the first time a unique case of NPL with an unusual presentation of oculomotor nerve palsy associated with pan-pituitary involvement in a diabetic patient. The incidence of cranial neuropathy in patients with diabetes should not merely be attributed to diabetic neuropathy without further evaluation.

## Data Availability

All data used during the current study are available from the corresponding author on reasonable request.

## Abbreviations

NPL: Nasopharyngeal lymphoma
NHL: Non-Hodgkin lymphoma
MRI: Magnetic resonance imaging
RBC: Red blood cell
WBC: White blood cell count
LDH: Lactate dehydrogenase
ACE: Angiotensin-converting enzyme
PPD: Purified protein derivative
P-ANCA: Perinuclear anti-neutrophil antibodies
IgG: Immunoglobulin G
C-ANCA: Cytoplasmic anti-neutrophil antibodies
TSH: Thyroid-stimulating hormone
ACTH: Adrenocorticotrophic, hormone
LH: Luteinizing hormone
FSH: Follicle-stimulating hormone
IGF-1: Insulin growth factor-1
CSF: Cerebrospinal fluid
IHC: Cell immunohistochemistry
HRCT: High-resolution computed tomography
CHOP: Cyclophosphamide, doxorubicin, vincristine, and dexamethasone
CDI: Central diabetes insidious
AVP: Arginine vasopressin
CRH: Corticotropin-releasing hormone
